# Neither NRT aided gradual cessation nor abrupt cessation is superior in producing long-term abstinence: Reconciling conflicting results from two recent meta-analyses

**DOI:** 10.18332/tid/113378

**Published:** 2019-11-15

**Authors:** Elias M. Klemperer, Nicola Lindson

**Affiliations:** 1 Department of Psychiatry, University of Vermont, Burlington, United States; 2 Nuffield Department of Primary Care Health Sciences, University of Oxford, Oxford, United Kingdom

**Keywords:** smoking cessation, gradual, abrupt, meta-analysis

**Dear Editor,**

Reduction in tobacco smoking before attempting to quit is common (i.e. gradual cessation)^1^, but guidelines for health professionals recommend abrupt smoking cessation^2,3^. We recently completed a Cochrane systematic review and meta-analysis including 22 trials (9219 participants) of gradual versus abrupt smoking cessation and found neither approach produced superior long-term cessation outcomes (RR=1.01; 95% CI: 0.87–1.17; I^2^=29%). We found similar results when restricting the analysis to 9 trials of gradual cessation versus abrupt cessation aided by nicotine replacement therapy (NRT) (RR=0.91; 95% CI: 0.72–1.16; I^2^=26%; 4359 participants)^4^. However, Tan et al.^5^ recently published a meta-analysis that concluded abrupt cessation was superior to gradual cessation with the aid of NRT, which contradicts our findings^4^. Tan et al.^5^ report that they restricted their meta-analysis to 3 trials where abstinence was biochemically verified and ‘both groups used an equal amount of NRT before and after quitting’. However, two of their three included trials provided NRT to participants before quitting in the gradual but not in the abrupt cessation condition^6,7^, and the third provided more NRT to the gradual than to the abrupt condition in the period before the quit day^8^. Our meta-analysis of NRT-aided studies included the three trials in the Tan et al.^5^ meta-analysis, plus an additional six trials comparing gradual versus abrupt smoking cessation^4^. Due to the design of the existing trials testing this comparison, neither meta-analysis provided a test of gradual versus abrupt cessation with equivalent pre- and post-quit NRT across conditions.

There are a number of methodological limitations to the Tan et al.^5^ review, two of which were likely to have led to the limited number of studies found eligible for inclusion. First, they only included studies that used biochemically verified abstinence. Although, this is a gold standard for measuring smoking abstinence in trials^9^, and its absence may inflate absolute abstinence rates, there is no apparent reason to assume that the likelihood of misreporting abstinence would differ between the gradual and abrupt conditions. Thus, the use of self-reported abstinence in some trials is unlikely to have affected relative abstinence rates (e.g. risk ratios) and should be included in meta-analyses to maximize the use of existing research. Second, they reported the use of very limited search terms ([‘smoking cessation’] AND [‘abrupt’ OR ‘gradual’]), which appear to have missed much of the relevant literature. Many relevant trials do not use the terms ‘gradual’ or ‘abrupt’ to describe their methodology. This is supported by the fact that their searches resulted in 134 records for screening^5^, as opposed to the 1944 records screened by Lindson et al.^8^. Thus, we conclude that due to methodological limitations, the Tan et al.^5^ findings do not fully represent existing research. When all trials of NRT-aided gradual versus abrupt smoking cessation are meta-analyzed, neither approach to quitting is superior in producing long-term abstinence^4^. Therefore, we conclude that people who do not wish to quit smoking abruptly could be encouraged to quit gradually.

## CONFLICTS OF INTEREST

The authors have completed and submitted the ICMJE Form for Disclosure of Potential Conflicts of Interest and none was reported.
